# The use of aortic balloon occlusion in traumatic shock: first report from the ABO trauma registry

**DOI:** 10.1007/s00068-017-0813-7

**Published:** 2017-08-11

**Authors:** M. Sadeghi, K. F. Nilsson, T. Larzon, A. Pirouzram, A. Toivola, P. Skoog, K. Idoguchi, Y. Kon, T. Ishida, Y. Matsumara, J. Matsumoto, V. Reva, M. Maszkowski, A. Bersztel, E. Caragounis, M. Falkenberg, L. Handolin, B. Kessel, D. Hebron, F. Coccolini, L. Ansaloni, M. J. Madurska, J. J. Morrison, T. M. Hörer

**Affiliations:** 1 Västmanlands Hospital Västerås, Department of Vascular Surgery, Örebro University, Örebro, Sweden; 2 Department of Cardiothoracic and Vascular Surgery, Faculty of Medicine and Health, Örebro University Hospital, 701 85 Örebro, Sweden; 3 Senshu Trauma and Critical Care Center, Rinku General Medical Center, Izumisano, Japan; 4 Emergency and Critical Care Center, Hachinohe City Hospital, Hachinohe, Japan; 5 Emergency and Critical Care Center, Ohta Nishinouchi Hospital, Koriyama, Japan; 6 Department of Emergency and Critical Care Medicine, Chiba University Graduate School of Medicine, Chiba, Japan; 7 R Adams Cowley Shock Trauma Center, University of Maryland, College Park, MD USA; 8 Department of Emergency and Critical Care Medicine, St Marianna University School of Medicine, Kawasaki, Japan; 9 Department of War Surgery, Kirov Military Medical Academy, Saint Petersburg, Russia; 10 Dzhanelidze Research Institute of Emergency Medicine, Saint Petersburg, Russia; 11 Sahlgrenska University Hospital, Department of Surgery, University of Gothenburg, Gothenburg, Sweden; 12 Department of Vascular Surgery, Örebro University, Örebro, Sweden; 13 Department of Radiology, Örebro University, Örebro, Sweden; 14 Helsinki University Hospital, Department of Orthopedics and Traumatology, University of Helsinki, Helsinki, Finland; 15 Department of Surgery, Hillel Yaffe Medical Centre, Hadera, Israel; 16 Department of Surgery, Papa Giovanni XXIII Hospital, Bergamo, Italy; 17 Department of Vascular Surgery, Queen Elizabeth University Hospital, Glasgow, UK

**Keywords:** Aortic occlusion, Trauma, REBOA, IABO, Hemorrhage

## Abstract

**Purpose:**

Resuscitative endovascular balloon occlusion of the aorta (REBOA) is a technique for temporary stabilization of patients with non-compressible torso hemorrhage. This technique has been increasingly used worldwide during the past decade. Despite the good outcomes of translational studies, clinical studies are divided. The aim of this multicenter-international study was to capture REBOA-specific data and outcomes.

**Methods:**

REBOA practicing centers were invited to join this online register, which was established in September 2014. REBOA cases were reported, both retrospective and prospective. Demographics, injury patterns, hemodynamic variables, REBOA-specific data, complications and 30-days mortality were reported.

**Results:**

Ninety-six cases from 6 different countries were reported between 2011 and 2016. Mean age was 52 ± 22 years and 88% of the cases were blunt trauma with a median injury severity score (ISS) of 41 (IQR 29–50). In the majority of the cases, Zone I REBOA was used. Median systolic blood pressure before balloon inflation was 60 mmHg (IQR 40–80), which increased to 100 mmHg (IQR 80–128) after inflation. Continuous occlusion was applied in 52% of the patients, and 48% received non-continuous occlusion. Occlusion time longer than 60 min was reported as 38 and 14% in the non-continuous and continuous groups, respectively. Complications, such as extremity compartment syndrome (*n* = 3), were only noted in the continuous occlusion group. The 30-day mortality for non-continuous REBOA was 48%, and 64% for continuous occlusion.

**Conclusions:**

This observational multicenter study presents results regarding continuous and non-continuous REBOA with favorable outcomes. However, further prospective studies are needed to be able to draw conclusions on morbidity and mortality.

## Introduction

Non-compressible torso hemorrhage (NCTH) is a challenge for trauma clinicians worldwide. Despite advances in the delivery of trauma care, such as damage control resuscitation (DCR) and the formalization of trauma systems, mortality from exsanguination remains as high as 45% [1]. Hemorrhage control and resuscitation are key management principles; however, these can be difficult to deliver in a timely manner and are often logistically complex.

Resuscitative endovascular balloon occlusion of the aorta (REBOA) is a technique whereby a compliant balloon is temporarily inflated in the aorta, thereby reducing distal blood flow and increasing cardiac afterload [2–4]. This hemodynamic profile is highly advantageous to bleeding patients; thus, REBOA has been proposed as a temporizing adjunct in the management of NCTH until definitive intervention can be achieved [5, 6].

However, this is controversial, as REBOA delivery requires specialist training, and balloon deflation can be associated with significant ischemia–reperfusion injury. The current evidence base is weak, with studies showing both good and bad outcomes, although data have often been drawn from registries conceived for different purposes or studies reporting small numbers of cases [7, 8].

In an effort to overcome these shortcomings, the Aortic Balloon Occlusion Trauma Registry (ABO Trauma Registry; www.abotraumaregistry.com) was established in 2014, with the goal of capturing REBOA-specific data from centers using this adjunct across the world. While still in a nascent form, the aim of the current study is to present the initial findings of the registry and patient outcomes.

## Methods

### Registry overview

The ABO Trauma Registry is designed to provide a mechanism for retrospective and prospective data capture for trauma patients in hemorrhagic shock, where management includes the use of REBOA. Center recruitment is ad hoc, with known REBOA-practicing institutions invited to participate directly, but centers can also register independently, via the registry website after approval from the investigators.

To capture clinically pragmatic data, there are no center-specific criteria, such as minimum case volume or hospital size. The registry is funded and hosted by the Department of Cardiothoracic and Vascular Surgery, Örebro University Hospital, Sweden.

### Ethical permission and data security

Prior to accepting cases, ethical approval was obtained from the regional committee (study number: 2014/210; Uppsala, Sweden). Participating centers also had to obtain approval from their local ethics committee.

Patient data are anonymized at the point of registration with a unique registry-generated ID number; no patient identifiable data, such as date of birth, are held. All data are held on a secure electronic database, and a secured password has been given to centers joining the registry to be able to enter data.

### Data collection

Collection is made via the website and includes data pertaining to demographics, mechanism of injury, vital signs and Glasgow coma scale (GCS) prehospital and on admission, laboratory tests, injury severity score (ISS), systolic blood pressure (SBP) before and after REBOA inflation, REBOA and non-REBOA interventions, puncture technique, speciality obtaining access, number of access attempts, zone of occlusion, occlusion time, and resuscitation.

Transfusion size per unit for blood products varies between countries. To be able to calculate the amount of transfusions, units were converted into Swedish transfusion sizes; 300 ml/packed red blood cells (PRBC), 250 ml/fresh frozen plasma (FFP) and 250 ml/platelets (PLT).

Further variable analysis was done on REBOA-specific data related to the length of occlusion, zones of occlusion and the REBOA technique employed. The zones of REBOA occlusion have been defined previously, but in brief: zone I extends from the origin of the left subclavian artery to the celiac trunk, zone II is from the celiac trunk to the lowest renal artery, and zone III involves the infrarenal aorta [3].

REBOA techniques have been defined as continuous (CO) or non-continuous (NCO). A CO technique is described as using a balloon fully inflated from insertion for the entire duration of its use, and only deflated once it is no longer required clinically. This is in contrast to NCO REBOA techniques, which are a heterogeneous group of techniques, such as partial or intermittent inflation. Partial REBOA (pREBOA) is where the balloon volume is reduced to permit a level of flow-through, whereas intermittent occlusion (iREBOA) is where the balloon is deflated entirely at regular intervals [9–12]. Both these techniques have been described as strategies to ameliorate ischemia–reperfusion injury. There is no universally accepted definition of these terms, so we have elected to describe this group as “non-continuous” REBOA techniques to avoid ambiguity.

Complication data were collected in relation to REBOA management, such as balloon migration/rupture, aortic rupture, puncture site hemorrhage, extremity compartment syndrome, and distal embolus, but there are also general complications, such as organ failure. Finally, patient outcome was defined as 30-day mortality.

### Data analysis

Continuous data are presented using mean ± standard deviation or median and interquartile range (25th–75th percentiles), depending on data distribution. Binary and nominal data are presented using numbers and proportions of available data due to missing information. A simple univariate comparison between survivors and fatalities was made; however, due to anticipated low numbers, multivariate analysis was not planned for this report. Continuous data were compared using a Student *t* test or Mann–Whitney *U* test, and categorical data using a Chi squared test. Paired* t* test and Wilcoxon’s test were used for comparison of pre- versus post-REBOA inflation. Significance was set at *p* < 0.05. Statistical analysis was completed using IBM SPSS version 23.0 (Armonk, NY, USA).

## Results

### Registry overview

**Fig. 1:**
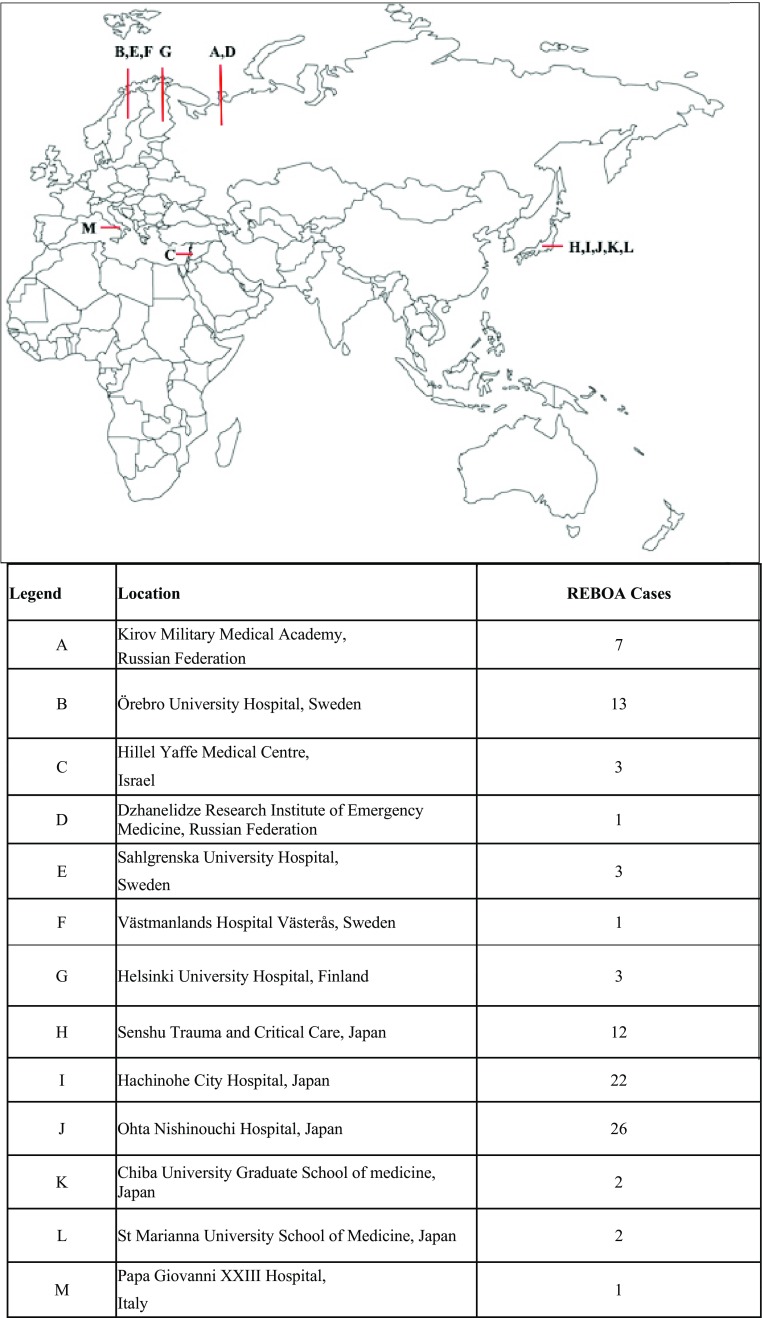
Location

Data were collected retrospectively from November 2011 and prospectively sampled from September 2014 until the end of September 2016. A total of 99 cases were registered: 38 prospective and 61 retrospective from 13 different hospitals within 6 countries (Fig. 1). However, 3 patients were excluded for having not met the inclusion criteria: REBOA was used in non-traumatic shock (*n* = 1), the balloon was not deployed due to access difficulties (*n* = 1), and the balloon was not inflated (*n* = 1). A total of 96 patients remained in the analysis (Table 1).

**Table 1:** Baseline demography, injury pattern, pre-hospital, ED and laboratory data for the cohorts

	All	Survivors	Fatalities	*P*
*N*	96	41	55	
Demography				
Age/years (total = 84)	52 ± 22	51 ± 20	54 ± 23	0.280
Age <60 years *n* (%) (total = 49)	49 (58%)	25 (64%)	24 (53%)	0.318
Age >60 years *n* (%) (total = 35)	35 (42%)	14 (36%)	21 (47%)	
Male/*n* (%) (total = 96)	65 (68%)	29 (69%)	36 (67%)	0.805
Pre-morbid health (total = 58)				
Co-morbid *n* (%)	27 (47%)	15 (60%)	12 (36%)	0.074
No co-morbidity *n* (%)	31 (53%)	10 (30%)	21 (64%)	
Injury mechanism (total = 95)				
Blunt/*n* (%)	84 (88%)	35 (85%)	49 (91%)	0.257
Penetrating/*n* (%)	9 (10%)	4 (10%)	5 (9%)	
Mixed/*n* (%)	2 (2%)	2 (5%)	0 (0%)	
Injury pattern and location				
ISS/median IQR (total = 82)	41 (29–50)	34 (20–42)	41 (34–51)	0.001
Thorax/*n* (%) (total = 96)	25 (26%)	4 (10%)	21 (39%)	0.001
Abdomen/*n* (%) (total = 96)	57 (59%)	26 (62%)	31 (57%)	0.656
Pelvis/*n* (%) (total = 96)	44 (46%)	19 (45%)	25 (46%)	0.918
Head injury/*n* (%) (total = 91)	36 (40%)	12 (30%)	24 (47%)	0.099
Pre-hospital data				
CPR/*n* (%) (total = 91)	18 (20%)	5 (13%)	13 (25%)	0.149
SBP <80 mmHg/*n* (%) (total = 50)	29 (58%)	10 (46%)	19 (68%)	0.111
SBP >80 mmHg/*n* (%) (total = 50)	21 (42%)	12 (55%)	9 (32%)	
GCS <8/*n* (%) (total = 75)	28 (37%)	7 (24%)	21 (46%)	0.061
GCS >8/*n* (%) (total = 75)	47 (63%)	22 (76%)	25 (54%)	
Emergency department data				
CPR/*n* (%) (total = 79)	11 (14%)	3 (9%)	8 (18%)	0.220
SBP <80 mmHg/*n* (%) (total = 65)	43 (66%)	19 (60%)	24 (73%)	0.255
SBP >80 mmHg/*n* (%) (total = 65)	22 (34%)	13 (41%)	9 (27%)	
Laboratory data on admission				
Hb/g/L/mean Std (total = 85)	10.0 ± 2.4	10.4 ± 2.3	9.6 ± 2.5	0.211
pH/median IQR (total = 76)	7.20 (7.05–7.34)	7.30 (7.14–7.39)	7.13 (6.99–7.27)	0.001
Base deficit/mean Std (total = 46)	−11.0 ± 9.3	−9.0 ± 5.7	−12.8 ± 11.6	0.038
Lactate/mmol/l/median IQR (total = 45)	8.1 (4.3–13.8)	6.6 (3.4–9.9)	10.6 (5.6–15.0)	0.066
INR/median IQR (total = 51)	1.3 (1.2–1.7)	1.2 (1.1–1.4)	1.5 (1.3–2.4)	0.001

Categorical data presented as numbers with proportions of cases for each column. Continuous data presented as median (interquartile range) or mean ± standard deviation depending on the distribution. The total number of reported cases for each variable is presented in parentheses in each row

### Patient characteristics, injury patterns and admission physiology

Sixty-four cases (67%) were reported from Japan. The mechanism of injury was blunt trauma in 88% (84/95) of the patients, and the mean age was 52 ± 22 yrs. Most of the patients (68%) were males without reported comorbidity (53%), with a median injury severity score (ISS) of 41 (IQR 29–50) (Table 1). The most common injuries were located in the abdomen (liver, spleen, major abdominal vessels) and pelvis.

Cardiopulmonary resuscitation (CPR) was performed at the injury site in 20% of the patients (18/91) and was ongoing through to the Emergency room (ER) in 14% (11/79) of the reported cases. The prehospital GCS score was >8 in 63% (47/75) of the cases, and 40% (36/91) were reported to have a head injury (Table 1). Pupillary response was absent in 10 out of 37 reported patients, and 90% of those patients died within 30 days. Intubation was performed in the ER in 64% (35/55) of the patients, and 90% (86/96) survived the ER (Table 1).

On admission, 66% (43/65) were in deep hemodynamic shock with a SBP of <80 mmHg. Median SBP before REBOA was 60 mmHg (IQR 40–80), which increased to 100 mmHg (IQR 80–128) immediately after balloon inflation in the hospital. SBP was higher in the survival group compared to the fatalities, before and after inflation (*p* < 0.05; Tables 1, 2).

**Table 2:** REBOA specific data

	All	Survivors	Fatalities	*P*
*n*	96	41	55	
Location of REBOA (total = 88)				
Emergency room/*n* (%)	58 (66%)	24 (63%)	34 (68%)	0.608
Operating room/*n* (%)	14 (16%)	6 (16%)	8 (16%)	
Hybrid operating room/*n* (%)	14 (16%)	6 (16%)	8 (16%)	
Intensive care unit/*n* (%)	1 (1%)	1 (3%)	0	
Other/*n* (%)	1 (1%)	1 (3%)	0	
SBP				
SBP before inflation/median IQR **(**total = 88)	60 (40–80)	70 (54–88)	50 (0–74)	0.006
SBP after inflation/median IQR (total = 89)	100 (80–128)	111(91–135)	95 (65–125)	0.029
Puncture technique (total = 85)				
Landmark guided/*n* (%)	71 (84%)	34 (92%)	37 (77%)	0.073
Ultrasound guided/*n* (%)	5 (6%)	1 (3%)	4 (8%)	
Fluoroscopy guided/*n* (%)	3 (3.5%)	2 (5%)	1 (2%)	
Cut-down/*n* (%)	6 (7%)	0	6 (13%)	
Specialty obtaining access (total = 90)				
ED/ICU/*n* (%)	60 (67%)	29 (74%)	31 (61%)	0.405
Vascular surgeon/*n* (%)	20 (22%)	8 (21%)	12 (24%)	
General surgeon/*n* (%)	3 (3%)	0	3 (6%)	
Radiologist/*n* (%)	6 (7%)	2 (5%)	4 (8%)	
Attempts at arterial access (total = 58)				
1/*n* (%)	32 (55%)	13 (54%)	19 (56%)	0.526
2–3/*n* (%)	21 (36%)	10 (42%)	11 (32%)	
>3/*n* (%)	5 (9%)	1 (4%)	4 (12%)	
Zone of occlusion (total = 92)				
Zone 1/*n* (%)	86 (94%)	37(93%)	49 (94%)	0.674
Zone 2/*n* (%)	3 (3%)	2 (5%)	1 (2%)	
Zone 3/*n* (%)	3 (3%)	1 (3%)	2 (4%)	
Total occlusion time (total = 73) [min/*n* (%)]				
<30	26 (36%)	15 (48%)	11 (26%)	0.147
31–60	26 (36%)	9 (29%)	17 (41%)	
>60	21 (29%)	7 (23%)	14 (33%)	
<40	38 (52%)	19 (61%)	19 (45%)	0.175
>40	35 (48%)	12 (39%)	23 (55%)	
Complications				
Balloon migration/*n* (%) (total = 90)	4 (4%)	3 (8%)	1 (2%)	0.208
Balloon rupture/*n* (%) (total = 90)	3 (3%)	1 (3%)	2 (4%)	0.751
Extremity compartment syndrome/*n* (%) (total = 42)	3 (7%)	3 (19%)	0	0.022
Signs of embolization/*n* (total = 85)	3 (4%)	1 (3%)	2 (4%)	0.657
Aortic/iliac rupture/*n* (total = 61)	0	0	0	
Access site bleeding/*n* (total = 82)	0	0	0	

### Methods and location of arterial access

In the majority of the cases (66%, 58/88), femoral access was achieved in the emergency room, followed by the operating room or the hybrid operating room to the same extents. The puncture method was blind puncture in 84% of cases (71/85), and was mostly conducted by emergency/intensive care physicians (67%, 60/90). Access was established on the primary attempt in 55% (32/58) of the cases. (Table 2). The most frequently used sheaths were 7 Fr (39%, 35/91) mostly used in Japan, followed by 10 and 11 Fr, used frequently in other countries.

### REBOA procedure

Zone I occlusion was used in 94% of patients (Table 2). Adjustment of the occlusion level was made in 34% of the 62 reported cases (for different reasons). There were 6 cases where adjustment was made to a different zone, mostly from zone I to III. One adjustment was due to balloon migration. The rest of the adjustments, 14 cases, were within the same zone.

### Methods of definitive hemostasis, resuscitation and critical care

Thoracotomy was performed in 29 patients of whom 21 underwent aortic clamping, with 86% mortality. Aortic clamping was performed before REBOA insertion in these cases. Laparotomy was performed in 43 patients, embolization in 42, and brain surgery in 15. No intervention could be made in 9 patients (Table 3).

**Table 3:** Data relating to imaging, modality of definitive hemorrhage control and resuscitation

	All	Survivors	Fatalities	*P*
*n*	96	41	55	
Disposal from ED (total = 96)				
Operating room/*n* (%)	25 (26%)	10 (24%)	15 (28%)	0.839
CT/*n* (%)	10 (10%)	6 (14%)	4 (7%)	
Angio suite/*n* (%)	4 (4%)	1 (2%)	3 (6%)	
ICU/*n* (%)	2 (2%)	1 (2%)	1 (2%)	
Hybrid operating room/*n* (%)	12 (13%)	6 (14%)	6 (11%)	
Hemorrhage control intervention				
Laparotomy/*n* (%) (total = 96)	44 (46%)	23 (55%)	21 (39%)	0.122
Thoracotomy/*n* (%) (total = 84)	29 (35%)	3 (9%)	26 (53%)	<0.001
Angioembolization/*n* (%) (total = 93)	42 (45%)	20 (48%)	22 (43%)	0.666
Pelvic external-fixation/*n* (%) (total = 51)	25 (49%)	13 (54%)	12 (44%)	0.488
Brain surgery/*n* (%) (total = 15)	15 (100%)	3 (20%)	12 (80%)	
No intervention/*n* (%) (total = 96)	9 (9%)	3 (7%)	6 (11%)	0.508
Resuscitation				
PRBC/median IQR (total = 81)	7 (3–14)	8 (4–12)	6 (2–14)	0.554
FFP/median IQR (total = 79)	8 (4–13)	8 (4–17)	8 (4–12)	0.413
PLT/median IQR (total = 74)	1 (0–2)	2 (1–2)	1 (0–2)	0.052
ICU care				
Days/median IQR (total = 73)	1 (0–5)	5 (2–7)	0 (0–1)	<0.001
Multiple organ failure/*n* (%) (total = 29)	10 (35%)	2 (18%)	8 (44%)	0.149
30-day mortality				
*n* (%) (total = 96)	54 (56%)	0		

The transfusion requirement for the first 24 h after REBOA placement shows a median of 7 U PRBC (IQR 3–14, 1 unit PRBC appr 300 ml), 8 U FFP (IQR 4–13, 1 unit FFP appr 250 ml), and 1 U PLT (IQR 0–2, 1 unit 250 ml) (Table 3).

### Continuous vs. non-continuous occlusion and complications

**Fig. 2:**
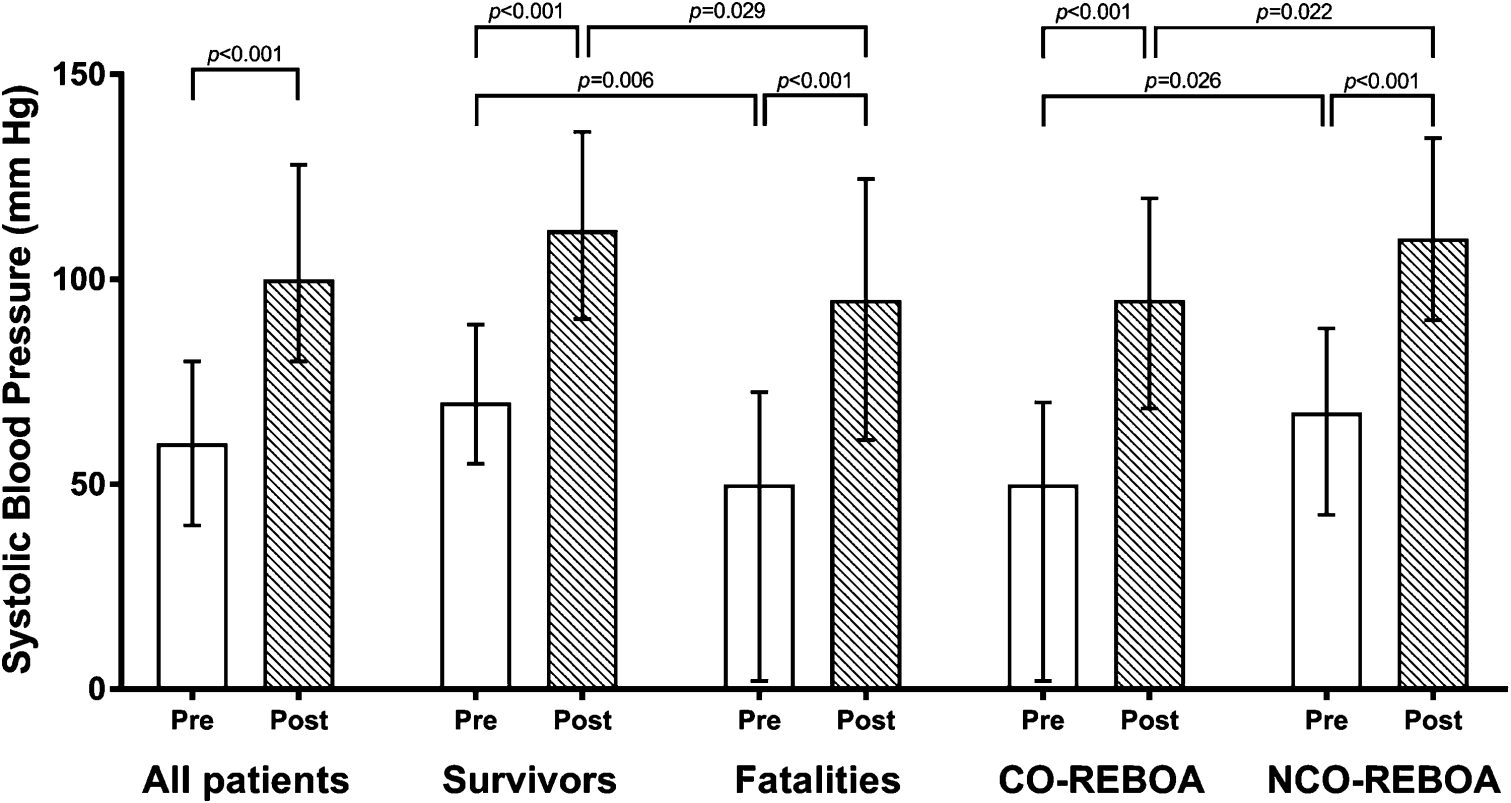
Pre- and post-REBOA systolic blood pressures for all patients (*n* = 88), survivals (*n* = 40), fatalities (*n* = 49), continuous-REBOA (CO-REBOA, *n* = 44) and non-continuous-REBOA (NCO-REBOA, *n* = 45)

The clinician in charge decided whether to use CO or NCO REBOA, probably according to the circulatory status of the patient. There were no significant differences between CO and NCO occlusion groups regarding demography, sex, mechanism of injury, ISS, prehospital GCS or occlusion time (Table 4). However, SBP before inflation and after inflation were higher in the NCO REBOA than in the CO REBOA group (*p* < 0.05) (Fig. 2).

**Table 4:** Comparison of continuous- to non-continuous REBOA

	Continuous REBOA	Non-continuous REBOA	*P*
n	50	46	
Demography			
Male/*n* (%) (total = 96)	35 (70%)	30 (65%)	0.617
Age/mean Std (total = 84)	54 ± 25	51 ± 18	0.403
Mechanism of injury (total = 95)			
Blunt/*n* (%)	42 (86%)	42 (91%)	0.365
Penetrating/*n* (%)	5 (10%)	4 (9%)	
ISS			
Median (IQR) (total = 82)	41 (30–54)	38 (26-50)	0.255
Occlusion Time (total = 73) [min/*n* (%)]			
<30	13 (46%)	13 (29%)	0.083
31–60	11 (39%)	15 (33%)	
>60	4 (14%)	17 (38%)	
Pre-hospital data			
GCS < 8/*n* (%) (total = 75)	13 (41%)	15 (35%)	0.611
CPR/*n* (%) (total = 91)	11 (23%)	7 (16%)	0.370
Systolic blood pressure			
ED Admission < 80 mmHg/*n* (%) (total = 65)	22 (67%)	21 (66%)	0.929
SBP mm Hg before inflation/median (IQR) (total = 88)	50 (0–70)	68 (43–88)	0.026
SBP mmHg after inflation/median (IQR) (total = 89)	95 (69–120)	110 (90–135)	0.022
Laboratory data on admission			
ED Lactate/median (IQR) (total = 45)	8.5 (4.2-13.0)	7.4 (4.5–13.8)	0.890
ED Base deficit/mean Std (total = 46)	−11.4 ± 10.0	−10.3 ± 8.2	0.426
ED INR/median (IQR) (total = 51)	1.4 (1.2–1.8)	1.2 (1.1–1.6)	0.078
Complications			
Extremity compartment syndrome/*n* (%) (total = 42)	3 (11%)	0.0	0.180
Balloon migration/*n* (%) (total = 90)	1 (2%)	3 (7%)	0.285
Balloon rupture/*n* (%) (total = 90)	1 (2%)	2 (5%)	0.531
Signs of embolization/*n* (%) (total = 85)	2 (4%)	1 (3%)	0.628
MOF/*n* (MOF/total) (total = 29)	6 (6/18)	4 (4/11)	0.868
Mortality			
*n* (%) (total = 96)	32 (64%)	22 (48%)	0.111

No major complications, such as bleeding from the REBOA access site or aorta perforation, were reported (Table 2). In the NCO group there were no reported cases with extremity compartment syndrome, but 3 cases were reported in the CO group. This was due to the sheath placement in 2 of the cases. Balloon migration and balloon rupture were reported in 5 cases in the NCO, and 2 cases in the CO group. Distal embolization was reported in 2 CO cases and 1 NCO case. There were 2 cases in the CO group with signs of distal embolization and 1 case in the NCO group. Multiple organ failure (MOF) was reported in 6 of 18 reported cases in the CO and 4 of 11 reported cases in NCO group (Table 4).

### Mortality

The median stay at the intensive care units was 5 days (IQR 2–7) for survivors and 0 days (IQR 0–1) for fatalities. The overall 30-day mortality was 56% (Table 3). There was no significant difference regarding mortality between patients older and younger than 60 years (Table 1). The 30-day mortality for the NCO group was 48%, and for the CO group 64% (Table 4).

## Discussion

The current study presents the initial 24-months experience of a global registry effort to assess the efficacy of aortic balloon occlusion in trauma patients. The registry reports a total of 96 patients recruited. The majority of the reported cases were blunt trauma with high ISS and age with a 30 days mortality of 64% for CO and 48% for NCO patients. This observational report presents interesting data on the management of extra-thoracic hemorrhage and non-compressible torso hemorrhage, and gives is the first registry data on continuous and non-continuous REBOA use.

The current study confirms and extends the findings of the only other published REBOA-specific registry, which is from North America. The prospective observational Aortic Occlusion for Resuscitation in Trauma and Acute Care Surgery (AORTA) demonstrated a mortality among REBOA patients (*n* = 46) of 72%, and among RT patients (*n* = 68) of 84% [13]. The AORTA registry aims to compare the two existing aortic occlusion techniques (REBOA vs. RT and aortic clamping) in traumatic and acute surgery patients in hemorrhagic shock. There are no prospective multicenter clinical studies with a large collection of patients described so far. The AORTA patients had a median ISS of 31; a majority were injured by a blunt mechanism (62%); and 60% were hypotensive (SBP <90 mmHg). They are similar to the patients in our cohort but with less blunt trauma (62 vs. 88%).

The development and evolution of these registries have been driven by a variety of factors, both experimental and clinical. Translational large-animal studies of REBOA have shown promising results in the management of hemorrhagic shock. REBOA has been shown to increase mean arterial pressure (MAP), decrease the hemorrhage, and reduce the fluid resuscitation volume. Histologic analysis shows a trend toward cardiac and visceral organ damage with occlusion time longer than 60 min [14–18]. In these studies, no aortic injury [14, 15] or necrosis of cerebral or spinal cord has been reported [17].

However, the clinical picture is less clear. The greatest experience worldwide is from Japan, where the Japanese National Trauma Databank (JTDB) has been capturing trauma data, including some REBOA data, for many years. Norii et al. and Inoue et al. have retrospectively compared REBOA and non-REBOA patients regarding mortality [19, 20]. These studies include a great number of patients (Norii *n* = 452) (Inoue *n* = 625) and adjusted the likelihood of receiving REBOA with propensity score analysis to match with non-REBOA patients. The studies conclude that REBOA is associated with higher mortality. Norii et al. calculated a mortality of 76% in REBOA patients. Inoue et al. reported an in-hospital mortality of 62% for REBOA versus 45% for non-REBOA patients.

The findings from these studies are also dependent upon the denominator. A further retrospective paper from Japan by Abe et al. [21] (*n* = 152) demonstrated that REBOA may indeed be superior to RT. The study’s endpoint was in-hospital mortality, which turned out to be 73% in REBOA patients and 91% in RT patients, respectively, similar to the AORTA study’s mortality. The authors mention differences in interventions performed in each group and the fact that the REBOA group had less severe thoracic injuries than the patients in the aortic cross-clamping group.

The Japanese studies from the JTDB have collected large cohorts. However, the JTDB is not REBOA or open aortic cross clamping oriented, and therefore lacks specific data related to aortic occlusion. The studies are retrospective and use propensity score matching and analysis to predict outcome after specific exposure. The risk with this methodology is that there are many confounding factors causing difficulty in interpreting the results.

Furthermore, the Japanese experience of the harmful effects of REBOA does not appear to be supported by data from North America and Europe. The clinical series from Gupta et al., Brenner et al. and Hörer et al. [2, 22, 23] have shown good outcomes in both penetrating and blunt trauma patients in hemorrhagic shock. But, despite good results these publications are clinical series with small groups of patients and low levels of evidence, which makes it difficult to reach any solid conclusions based on their observations.

The most interesting part of the current study is the presentation of non-continuous occlusion data. The current study demonstrates a reduced mortality, albeit not statistically significant, in patients treated with partial or intermittent techniques. The non-continuous aortic occlusion techniques show a generally lower mortality rate but also a lower morbidity rate regarding organ failure which is the cause of late deaths occurring in trauma patients [24]. Notably, compartment syndrome only occurred in the CO group. Theoretically, pREBOA has the effect of ameliorating reperfusion injury. This is potentially a very important finding and further research is required to identify the optimum method for pREBOA.

To date, there are not many clinical studies conducted about partial occlusion, albeit case and technical reports [10, 12]. The translational studies suggest that partial occlusion has the potential of reducing ischemic injuries [11, 25]. The results from non-continuous occlusion in our study should be carefully interpreted since there was a difference between the continuous and non-continuous groups in SBP before inflation of the balloon. In addition, estimation of the degree of the actual partial occlusion requires the possibility of measuring SBP above and below the occlusion site or blood flow distal to the balloon.

The registry faces a number of limitations, which should be discussed. With the current iteration of this registry, the focus has been on REBOA-specific data. Therefore, there is no comparison group to evaluate the efficacy of REBOA use, and there could be selection bias in the reported cases as there are no means for control of inclusion and exclusion of patients. It is also noted that there are missing data for some of the presented parameters. Other identified limitations are differences in indications/policies for REBOA use at each center since there are no defined specifications for its use. A longer follow-up of morbidity and late mortality (3–6 months) is another modification that can be made in future studies.

Some of the shortcomings listed above will be addressed via modifications to the registry in the case of further studies. Equally, a randomized control trial is due to start in the UK in 2017 (the UK-REBOA Trial), which should complement some of the experiences in this early report.

The ABO trauma registry is an effort to gather international data on the current clinical use of REBOA in traumatic shock. The registry gives an insight into international practice from a real-world perspective. Its data collection intends to be ongoing so further centers are encouraged to join and submit data.

It is important to emphasize that REBOA is an endovascular technique, which requires specific training for its management, and that it is a tool for temporary hemodynamic control until definitive repair. REBOA is therefore part of a trauma management/system, i.e. Endovascular Hybrid Trauma and Bleeding Management (EVTM) and should be used carefully [2]. In several countries, there are REBOA management courses but there are no standardized courses provided yet [26–28]. With the range of specialties involved in this procedure, it is now time to provide structured standardized courses such as the DCR.

## Conclusion

The first 24 months of the ABO registry marks a major achievement in establishing a mechanism for data collection. Further work is required to address some of the limitations identified in this preliminary analysis. However, overall, there is accumulating evidence that REBOA is an effective adjunct in the control of traumatic hemorrhage, and that non-continuous occlusion could be an alternative in particular cases. This adjunct can be delivered safely by a multi-disciplinary team with a low rate of procedural complications. Further statistical power in the registry is required before comments can be made regarding mortality.

## Abbreviations

NCTH: Non-compressible torso hemorrhage
DCR: Damage control resuscitation
REBOA: Resuscitative endovascular balloon occlusion of the aorta
PRBC: Packed red blood cells
FFP: Fresh frozen plasma
PLT: Platelets
CPR: Cardiopulmonary resuscitation
GCS: Glasgow coma scale
SBP: Systolic blood pressure
ISS: Injury severity score
ER: Emergency room
MOF: Multiple organ failure
MAP: Mean arterial pressure
pREBOA: Partial resuscitative endovascular balloon occlusion of the aorta
iREBOA: Intermittent resuscitative endovascular balloon occlusion of the aorta
CO: Continuous occlusion
NCO: Non-continuous occlusion
